# A Significant but Rather Mild Contribution of T286 Autophosphorylation to Ca^2+^/CaM-Stimulated CaMKII Activity

**DOI:** 10.1371/journal.pone.0037176

**Published:** 2012-05-16

**Authors:** Steven J. Coultrap, Kelsey Barcomb, K. Ulrich Bayer

**Affiliations:** Department of Pharmacology, University of Colorado Denver – School of Medicine, Aurora, Colorado, United States of America; Institute for Interdisciplinary Neuroscience, France

## Abstract

**Background:**

Autophosphorylation of the Ca^2+^/calmodulin (CaM)-dependent protein kinase II (CaMKII) at T286 generates partially Ca^2+^/CaM-independent “autonomous” activity, which is thought to be required for long-term potentiation (LTP), a form of synaptic plasticity thought to underlie learning and memory. A requirement for T286 autophosphorylation also for efficient Ca^2+^/CaM-stimulated CaMKII activity has been described, but remains controversial.

**Methodology/Principal Findings:**

In order to determine the contribution of T286 autophosphorylation to Ca^2+^/CaM-stimulated CaMKII activity, the activity of CaMKII wild type and its phosphorylation-incompetent T286A mutant was compared. As the absolute activity can vary between individual kinase preparations, the activity was measured in six different extracts for each kinase (expressed in HEK-293 cells). Consistent with measurements on purified kinase (from a baculovirus/Sf9 cell expression system), CaMKII T286A showed a mildly but significantly reduced rate of Ca^2+^/CaM-stimulated phosphorylation for two different peptide substrates (to ∼75–84% of wild type). Additional slower CaMKII autophosphorylation at T305/306 inhibits stimulation by Ca^2+^/CaM, but occurs only minimally for CaMKII wild type during CaM-stimulated activity assays. Thus, we tested if the T286A mutant may show more extensive inhibitory autophosphorylation, which could explain its reduced stimulated activity. By contrast, inhibitory autophosphorylation was instead found to be even further reduced for the T286A mutant under our assay conditions. On a side note, the phospho-T305 antibody showed some basal background immuno-reactivity also with non-phosphorylated CaMKII, as indicated by T305/306A mutants.

**Conclusions/Significance:**

These results indicate that Ca^2+^/CaM-stimulated CaMKII activity is mildly (∼1.2–1.3fold) further increased by additional T286 autophosphorylation, but that this autophosphorylation is not required for the major part of the stimulated activity. This indicates that the phenotype of CaMKII T286A mutant mice is indeed due to the lack of autonomous activity, as the T286A mutant showed no dramatic reduction in stimulated activity.

## Introduction

CaMKII is a ubiquitously expressed Ser/Thr kinase, with at least one of its four isoforms (α, β, γ, δ; encoded by different genes) found in any tissue examined, but with the highest expression level found in brain, where CaMKIIα alone constitutes 1% or more of total protein in the hippocampus and the cortex [1]–[3]. CaMKII is best known for its involvement in regulating forms of synaptic plasticity underlying higher brain functions such as learning and memory (for review see [4]–[6]). For instance, long-term potentiation (LTP) of synaptic strength requires CaMKII activity [7]–[12]. CaMKII activity is stimulated by Ca^2+^/CaM (∼1000fold), but LTP induction additionally requires a form of Ca^2+^/CaM-independent “autonomous” CaMKII activity [13], [14] that is generated by T286 autophosphorylation [15]–[18]. Interestingly, T286 autophosphorylation is, like LTP, more readily induced by high frequency stimulation [19]–[21]. However, contrary to common perception, T286 phosphorylated “autonomous” CaMKII is not fully active but is instead significantly further stimulated by Ca^2+^/CaM, at least for regular “R-substrates” [18]. Higher levels of autonomy (the ratio of autonomous over maximal stimulated kinase activity) were seen only for special substrates and required a special mechanism (additional binding to the CaMKII T-site by “T-substrates”) [18]. The default mechanism of further Ca^2+^/CaM-stimulation of autonomous CaMKII still allows for a “molecular memory” of past Ca^2+^ signals, but additionally prevents complete uncoupling from subsequent cellular Ca^2+^-stimuli [18].

T286 autophosphorylation occurs as an inter-subunit reaction within the 12meric CaMKII holoenzymes. This requires CaM binding not only to the subunit acting as kinase but also to the subunit acting as substrate (in order to make T286 accessible for phosphorylation) [22], [23], and this dual requirement is thought to underlie the frequency detection of Ca^2+^ oscillations by CaMKII [19]–[21]. Maximal Ca^2+^/CaM stimuli (>10fold activation constant of ∼100 nM Ca^2+^/CaM for CaMKIIα) induce T286 autophosphorylation with fast kinetics (∼12 sec^−1^ at 30°C) [24]. Thus, in assays of stimulated CaMKII activity, the initially naïve CaMKII becomes quickly autophosphorylated at T286 (within the first few seconds), and most of the activity measured by substrate phosphorylation is consequently mediated not by naïve but by T286-phosphorylated CaMKII (even when relatively short reaction times of <1 min are used). Indeed, several studies have described T286 phosphorylation as a pre-requisite for efficient Ca^2+^/CaM-stimulated substrate phosphorylation [25]–[28]. However, other studies indicated a requirement of phospho-T286 exclusively in autonomous but not in Ca^2+^/CaM-stimulated substrate phosphorylation [29]–[31].

Here, the substrate phosphorylation rates of CaMKII wild type and its T286A mutant were compared directly in order to determine the relative contribution of T286 autophosphorylation to stimulated CaMKII activity. The results indicate a significant but very modest (∼15–25%) contribution of T286 phosphorylation to maximal Ca^2+^/CaM-stimulated CaMKII activity. Beyond mere interest in the detailed regulation of an important signaling molecule, these findings support the interpretation that the phenotype of CaMKII T286A mutant mice (which show impaired LTP and learning) [13] is indeed due specifically to the lack of autonomous CaMKII activity (and not to a dramatic reduction of all forms of CaMKII activity).

## Materials and Methods

### Material

CaM and CaMKIIα was purified after bacterial or baculovirus/Sf9 cell expression [32]–[34]. The purification (as well as CaMKII activity assays and detection of CaMKII autophosphorylation) are described and discussed in detail elsewhere [34]. Substrate peptides were purchased from Genescript. Chemicals were obtained from Sigma and Perkin Elmer.

### Cell extracts and protein concentration

HEK 293 cells were transfected with GFP-CaMKIIα expression vectors by the calcium phosphate method (on 10 cm plates, 1 day after a 1∶5 split from confluent plates), and harvested 72 h later. Cells were rinsed, scraped into ice-cold PBS, and collected by low speed centrifugation (5 min at 1,000 g at 4°C). Then, the cells were homogenized with a motorized pellet pestle (Kontes) for 10 sec in 0.4 ml ice cold 50 mM PIPES pH 7.2, 10% glycerol, 1 mM EDTA, 1 mM DTT, and complete protease inhibitor (Roche). Debris was removed by centrifugation (20 min at 16,000 g at 4°C). A total of 12 CaMKII extracts were prepared in two separate rounds, with 3 extracts of each CaMKII wild type and T286A prepared in parallel in each individual round. CaMKII concentration was determined by their GFP-fluorescence measured in a spectrofluorometer (Fluoromax3; Horiba Jobin Yvon) at 488 nm excitation and 510 nm emission wavelength (based on at least three samplings with increasing extract concentrations). This method allows a very accurate comparison of the relative CaMKII concentrations in the different extracts; absolute concentrations were derived by comparison to a GFP-CaMKII standard (with its concentration determined by quantitative Western analysis [18]). The CaMKII concentrations in all extracts were within a three-fold range (1.12–3.68 µM subunits).

### CaMKII activity assays

**Figure 1:**
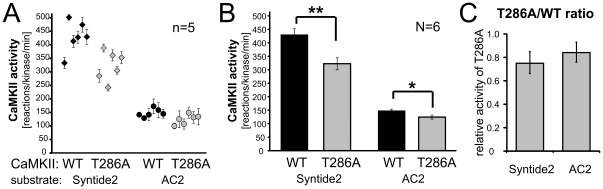
Maximal stimulated activity of CaMKII wild type and its T286A mutant was induced by 1 µM Ca^2+^/CaM and measured by the phosphorylation rate of two different substrates (syntide 2 and AC2) in biochemical assays. Error bars indicate mean ± s.e.m; **: p<0.01, *: p<0.05 in two-tailed t-test. *A*, CaMKII activity in the different extracts for each kinase form (n = 5 individual assays). *B*, The average activity of CaMKII from the individual extracts shown separately in panel A (N = 6 different extracts). *C*, The relative activity of the T286A mutant (compared to wild type activity normalized as 1 for each substrate) based on the results shown in panel B.

**Figure 2:**
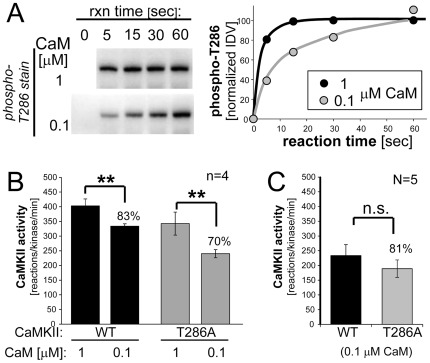
Submaximal stimulated activity of CaMKII wild type and its T286A mutant was induced by 0.1 µM Ca^2+^/CaM and measured by the phosphorylation rate of the substrate syntide 2 (in 1 min reactions at 30°C). Error bars indicate mean ± s.e.m; **: p<0.01, n.s.: p>0.05 in two-tailed t-test. *A*, T286 autophosphorylation of CaMKII wild type was assessed by Western analysis (left), and quantified by arbitrary relative immuno-detection values (IDV; right). T286 autophosphorylation stimulated by 0.1 µM Ca^2+^/CaM was slower compared to stimulation by 1 µM Ca^2+^/CaM, but the same level of maximal autophosphorylation was still achieved within 1 min reaction time at 30°C. *B*, Submaximal activation by 0.1 µM Ca^2+^/CaM was verified by comparing one individual preparation of each CaMKII wild type and T286A mutant to the activity induced by 1 µM Ca^2+^/CaM (n = 4 individual assays). *C*, The average activity of CaMKII from multiple preparations (N = 5) stimulated by 0.1 µM Ca^2+^/CaM did not differ significantly between CaMKII wild type and the T286A mutant. While the ratio of the mean activities of T286A over wild type was similar as observed at maximal stimulation (compare Fig. 1B,C), the variability at submaximal stimulation was greater (with standard deviations of 35–36% of the mean at submaximal stimulation compared to 13–17% at maximal stimulation for syntide 2 substrate).

Kinase activity assays (1 min at 30°C) were started by adding CaMKIIα (2.5 nM subunits) to a reaction mix containing 50 mM PIPES pH 7.2, 0.1% BSA, 1 µM CaM, 1 mM CaCl_2_, 10 mM MgCl_2_, 100 µM [γ-^32^P]ATP (∼1 Ci/mmole), 1 µM okadaic acid, and 75 µM substrate peptide (syntide 2 or AC2, as indicated). Under these conditions (used in Fig. 1), the Ca^2+^/CaM-stimulus and the substrate concentration are at or near saturation, while the chosen reaction time is within the linear range [18]. Additionally, submaximal Ca^2+^/CaM-stimuli (0.1 µM CaM) were used where indicated (in Fig. 2). Reactions were stopped by spotting the mix onto P81 cation exchange chromatography paper (Whatman) squares. After extensive washes in water, phosphorylation of the substrate peptide bound to the P81 paper was measured by the Cherenkov method.

### CaMKII autophosphorylation at T286 and T305

CaMKII phosphorylation at T286 and T305 was assessed by Western-analysis with phospho-selective antibodies (PhosphoSolutions) as described [18], [35]. The T286 phosphorylation state was assessed after reactions essentially as in the kinase activity assays (but with unlabeled ATP and without substrate peptide, and after different reaction times in presence of either 1 µM or 0.1 µM CaM as indicated). The T305 phosphorylation state was assessed after two different reaction schemes: (i) after prior CaMKII T286 autophosphorylation on ice followed by chelation of Ca^2+^ with EGTA (and with subsequent reaction times and temperatures as indicated), or (ii) after reactions with naïve CaMKII essentially as in the kinase activity assays (with 1 µM CaM but with unlabeled ATP and without substrate peptide).

## Results

### T286A mutant CaMKII shows lower Ca^2+^/CaM-stimulated activity

We have previously described the mechanisms underlying low CaMKII autonomy that is significantly further CaM-stimulated for regular “R-substrates” (such as syntide 2) compared to higher autonomy that is only mildly further Ca^2+^/CaM-stimulated for T-site binding “T-substrates” (such as AC2) [18]. As part of that study, we have ruled out differential effects of T286 autophosphorylation on the rate of Ca^2+^/CaM -stimulated phosphorylation of syntide 2 compared to AC2 as an underlying mechanism; for both CaMKII wild type and for its T286A mutant, Ca^2+^/CaM-stimulated syntide 2 phosphorylation occurred with an approximately 2fold higher Vmax compared to AC2 phosphorylation [18]. The results were reported with the activity of CaMKII wild type and T286A towards syntide 2 each normalized as 100%, in order to correct for differences between the individual purified kinase preparations [18], which can yield different levels of activity per mg of purified kinase. Indeed, the two preparations differed in the maximal CaM-stimulated activity, with the activity of the T286A mutant ∼55% of the wild type activity for both substrates tested.

In order to distinguish if the different activity of the purified CaMKII wild type versus T286A mutant was due to the individual kinase preparations or was instead caused by the mutation, we directly compared the activity of the two different kinase forms in six different extracts for each kinase (Fig. 1A). Again, lower CaM-stimulated phosphorylation rates were measured for the CaMKII T286A mutant compared to wild type (Fig. 1B), indicating that there is indeed a difference directly caused by the mutation. Such reduced phosphorylation rates by the T286A mutant were found both for syntide 2 (to ∼75%) and for AC2 (to ∼84%) (Fig. 1C). This reduction was statistically significant for both substrates (Fig. 1B), and statistically indistinguishable between them (Fig. 1C). However, the extent of the effect was rather mild, with only ∼1.2–1.3fold higher activity for CaMKII wild type compared to the T286A mutant. This indicates that the slightly larger difference initially seen between the two individual purified CaMKII wild and T286A mutant preparations (∼1.8fold) was caused in part by variability among individual preparations. Such variability was indeed observed among the six different individual preparations for CaMKII wild type (up to ∼1.5fold) as well as T286A (up to ∼1.6fold; see Fig. 1A).

Next, we decided to compare the activity of CaMKII wild type and the T286A mutant also at submaximal stimulation (0.1 µM instead of 1 µM CaM) using syntide 2 as substrate. As expected, T286 autophosphorylation of CaMKII wild type was slower at such submaximal stimulation, but still reached the same level within the 1 min reaction time (Fig. 2A). Submaximal stimulation was also verified by direct comparison of one individual kinase preparation for each CaMKII wild type and the T286A mutant with the different CaM concentrations in parallel assays (Fig. 2B). Compared to 1 µM CaM, activity stimulated by 0.1 µM CaM was ∼83% for CaMKII wild type and ∼70% for the T286A mutant (Fig. 2C). We reasoned that the difference between CaMKII wild type and the T286A mutant may become more obvious at submaximal stimulation. Contrary to this prediction, when five different extracts of the two CaMKII forms were compared after stimulation by 0.1 µM CaM, the mean activity of T286A compared to wild type remained reduced to a very similar level (∼81%; Fig. 2C) as seen with 1 µM CaM (∼75%; compare Fig. 1B,C). However, at submaximal stimulation, the variability was greater and the apparent difference between CaMKII wild type and the T286A mutant was no longer statistically significant (Fig. 2C).

In summary, the significant but mild (∼1.2–1.3fold) contribution of T286 autophosphorylation to stimulated activity under maximally Ca^2+^/CaM-stimulated conditions was not enhanced by submaximal Ca^2+^/CaM-stimulation conditions.

### The T286A mutant shows less T305 autophosphorylation

**Figure 3:**
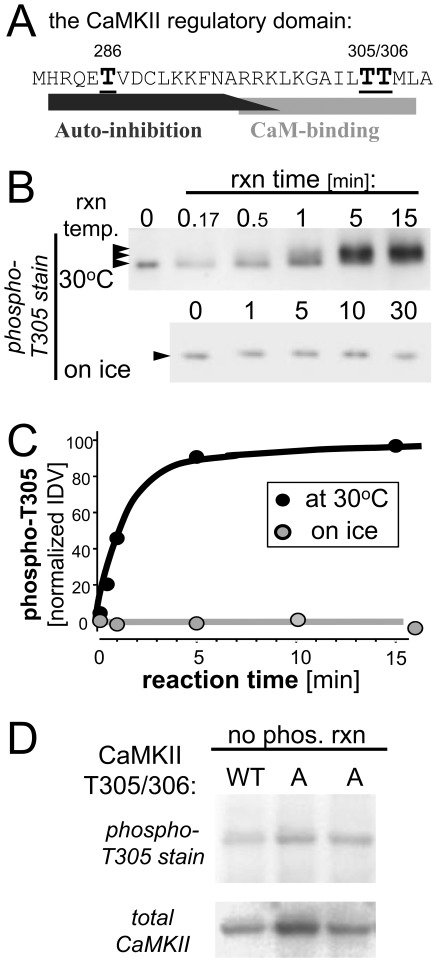
CaMKII T305/306 “burst” auto-phosphorylation *in vitro*. *A*, The sequence of the CaMKII regulatory domain with T286 in the autoinhibitory region and T305/306 in the CaM-binding region indicated. *B*, T305/306 phosphorylation was assessed by Western analysis. CaMKII was pre-phosphorylated at T286 on ice; the “burst” was induced by EGTA addition at 30°C or on ice, and stopped after different reaction times. Note the band-shift caused by phosphorylation of additional sites during the “burst” at 30°C, and the basal immuno-detection without phosphorylation reaction. *C*, Quantification of the relative T305/306 phosphorylation during the “burst” by arbitrary relative immuno-detection values (IDV). Basal immuno-detection prior to the phosphorylation reactions was subtracted in the quantification shown. No increase in phosphorylation was detected on ice. For 30°C, the results indicate an initial phosphorylation rate (at reaction times of 1 min or less) of ∼0.45 min^−1^ (assuming that saturation represents near-complete phosphorylation; otherwise the rate is even lower). *D*, The basal immuno-detection with the phospho-T305 antibody prior to the phosphorylation reactions was likely due to background immuno-reactivity, as indicated by comparison to T305/306A mutant CaMKII.

In order to explain the rational of our next experiments, several background issues need to be addressed first. The T305/306 residues are located within the Ca^2+^/CaM-binding region of the CaMKII regulatory domain (Fig. 3A). T305/306 autophosphorylation interferes with Ca^2+^/CaM binding and, *vice versa*, Ca^2+^/CaM binding is thought to interfere with this inhibitory autophosphorylation (for review see [5]). Autophosphorylation of T306 (but not T305) can be mediated by CaMKII in its basal state without any activation [36]. However, T305 and T306 autophosphorylation is induced most efficiently by dissociating Ca^2+^/CaM from T286 phosphorylated “autonomous” CaMKII (for instance by chelating Ca^2+^ with EGTA, as done in Fig. 3B,C), in a reaction that has been termed “burst” phosphorylation [37]. Compared to the fast T286 phosphorylation (see Fig. 2A; ∼12 sec^−1^ at 30°C [24] and near maximal phosphorylation within 0.5 min even on ice [38]), T305 “burst” phosphorylation (i.e. after addition of EGTA to T286 phosphorylated CaMKII) was dramatically slower (Fig. 3B,C), indicating a rate of ∼0.01 sec^−1^ during the linear phase at 30°C (here up to 1 min) and with no appreciable phosphorylation on ice even after 30 min (Fig. 3C). Previous studies that used the effect on stimulated CaMKII activity as a readout indicated similar rates for the T305/T306 burst phosphorylation (∼0.01–0.05 sec^−1^, based on the time observed for half-maximal kinase inhibition) [37], [39], [40], and found identical rates for T305 compared to T306 phosphorylation [37]. Note that the autophosphorylation induced by adding EGTA to T286-phosphorylated CaMKII causes a band-shift in the Western analysis (see Fig. 3B), and that this “burst” autophosphorylation includes additional sites (such as S314), but that only phosphorylation at T305 or T306 inhibits Ca^2+^/CaM-stimulation [37]. Also note that the phospho-antibody is directed only against T305 and showed significant immuno-reactivity also with non-phosphorylated CaMKII (10–20%), but was significantly further increased during phosphorylation reactions at 30°C (but not on ice; see Fig. 3B,C). The basal immuno-reactivity did not appear to be due to residual basal T305 phosphorylation in our CaMKII preparations, as indicated by comparison to T305/306A mutant CaMKII (Fig. 3D), consistent with a previous report of basal immuno-reactivity remaining also after phosphatase treatment [41]; this background was subtracted in the quantification of phosphorylation.

**Figure 4:**
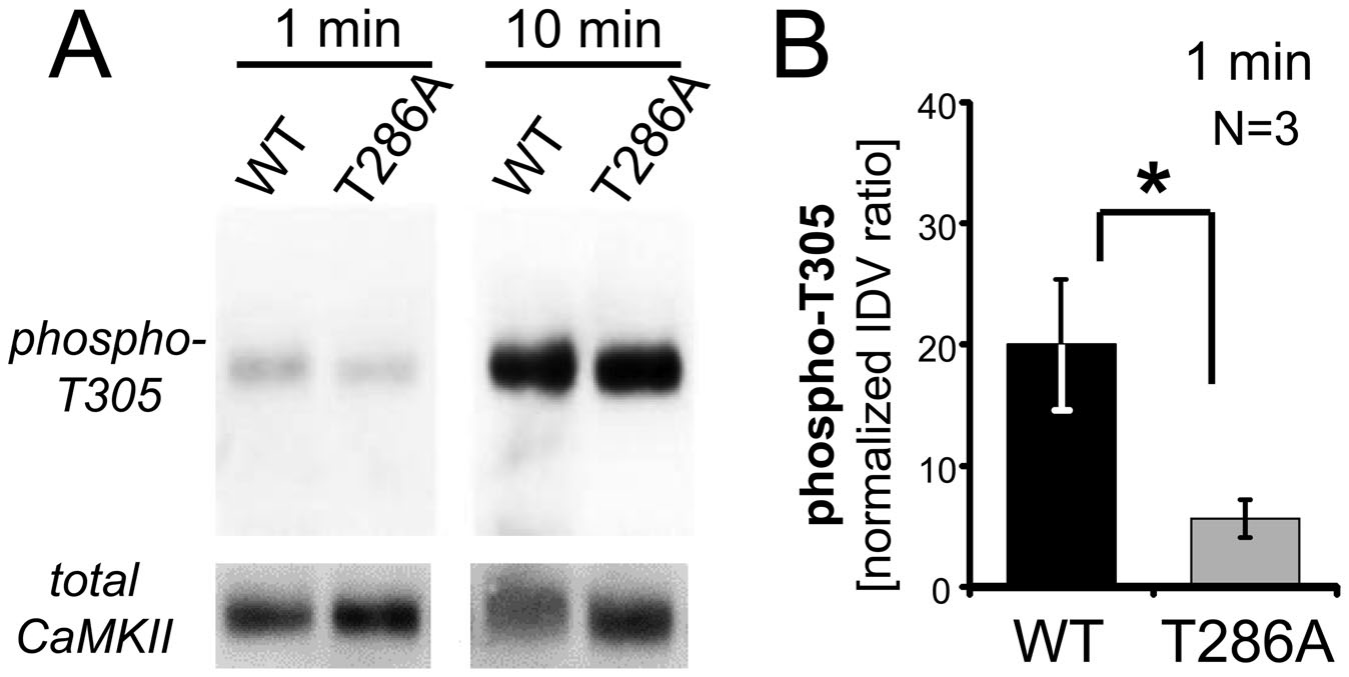
CaMKII T305 phosphorylation is further reduced for the T286A mutant. *A*, T305 phosphorylation was detected by Western analysis after reactions corresponding to the kinase activity assays shown in Fig. 1 (1 min at 30°C, but without substrate peptide) or after extended reaction times (10 min). *B*, Quantification of the relative T305 phosphorylation by the ratio of the phospho-T305 and total CaMKII immuno-detection values (IDV) shows significantly lower phosphorylation of the T286A mutant in our 1 min kinase assay conditions (N = 3 different extracts for each kinase form; *: p<0.05 in two-tailed t-test). The IDV ratio for T286A was ∼28% (±7.6%) of wild type after the 1 min reactions; in the 10 min reaction experiment shown in panel A, this ratio was ∼83%, consistent with the faster wild type reaction reaching saturation faster.

For CaMKII wild type, suppression of T305/306 phosphorylation by Ca^2+^/CaM during 1 min stimulated activity assays prevents significant contribution of this inhibitory phosphorylation to the kinase activity measured [18], [37], [39], [40]. Nevertheless, under our kinase activity assay conditions (but with substrate omitted), up to ∼20% of CaMKII wild type may become autophosphorylated at T305 after 1 min (which is reduced to ∼2–10% in the presence of substrate peptides) [18]. By contrast, T286 should be nearly maximally phosphorylated within the first few seconds of the reaction [24]. We reasoned that in the presence of Ca^2+^/CaM (which suppresses T305/306 phosphorylation), phospho-T286 may suppress T305/306 phosphorylation even further, as it slows down the Ca^2+^/CaM off-rate from ∼130 min^−1^ to <0.06 min^−1^, which should prevent any appreciable un-binding of CaM in the presence of Ca^2+^ (“CaM trapping”) [42]. Thus, we decided to test if the T286A mutant shows enhanced T305 autophosphorylation, which could explain its reduced Ca^2+^/CaM-stimulated activity. Contrary to this prediction, in a direct comparison to CaMKII wild type, the T286A mutant instead showed even less T305 autophosphorylation (less than one third compared to wild type) during 1 min reactions at 30°C (Fig. 4A,B). This indicates that T286 autophosphorylation does not contribute to the suppression of T305/306 autophosphorylation under Ca^2+^/CaM-stimulated conditions, and thus does not explain the mild reduction in kinase activity seen for the T286A mutant CaMKII in Fig. 1.

## Discussion

The results of this study indicate that CaMKII autophosphorylation at T286 (which generates Ca^2+^/CaM-independent “autonomous” CaMKII activity [15]–[18]) significantly but rather mildly (∼1.2–1.3fold) also further increases the maximal Ca^2+^/CaM-stimulated activity of CaMKII. This was done by directly comparing multiple preparations of CaMKII wild type and its T286A mutant for their phosphorylation rate, using two different substrates. Previous studies either described T286 autophosphorylation as a pre-requisite also for efficient Ca^2+^/CaM-stimulated CaMKII activity [25]–[28], or found no contribution of T286 to this stimulated activity at all [29]–[31]. These opposing conclusions impact not only our understanding of a regulatory detail of an important signaling enzyme, but also the interpretation of the phenotype of CaMKII T286A mutant mice [13], [43]–[47]. These mice show impaired LTP and learning [13], and our findings support the common interpretation that this is indeed due to the specific lack of autonomous CaMKII activity. A dramatic contribution of T286 also to stimulated activity would not allow conclusions about the specific form of CaMKII activity involved, and thus provide no additional information compared to the kinase dead CaMKII K42R mutant mice (which indeed also show impaired LTP and learning, as expected [48]) or to studies with CaMKII inhibitors (which, again, impair LTP [7], [14], [49]–[51] and learning [14]). But what caused the fundamentally different conclusions in the various biochemical studies? Most studies appeared to compare only individual CaMKII preparations, which can differ significantly in their activity (as was also seen here). This included a study that reported a ∼6fold higher activity of CaMKII wild type compared to the T286A mutant [28], using myosin light chain as substrate (which has a ∼12fold higher Km compared to syntide 2 [52]). Even without any change in the principle state of CaMKII activation, T286 phosphorylation may influence the interaction of CaMKII with some specific substrates, which would in turn effect their phosphorylation. Indeed, T286 phosphorylation can regulate CaMKII binding to several proteins (for review see [53], [54]), although binding to myosin light chain has to our knowledge not been examined. Two other studies reached their conclusion not based on comparison to a T286A mutant, but based on the observation that T286 autophosphorylation preceded substrate phosphorylation, with a lag-phase of substrate phosphorylation seen for naïve but not for T286 pre-phosphorylated CaMKII [25], [26]. As T286 autophosphorylation occurs very fast [24], it should indeed precede substrate phosphorylation even if it is not a principle requirement for the Ca^2+^/CaM-stimulated activity. Additionally, the lag-phase was observed only at ATP concentrations ∼2000fold lower than seen within cells (2 µM compared to cellular ∼4 mM). Under our assay conditions (100 µM ATP; ∼12fold its Km for CaMKII), no appreciable lag-phase is observed [18]. Another study found dramatically reduced Ca^2+^/CaM-stimulated activity for a T286A mutant, but again only at the low 2 µM nucleotide concentration (using ATPγS) [27]. At 50 µM ATP, the reported difference between the CaMKII wild type and T286A preparation was only ∼1.3fold, the same mild effect as observed here. Other studies instead found no difference between CaMKII wild type and several T286 mutants regarding Ca^2+^/CaM-stimulated CaMKII activity [29], [30] or even a mild increase [31]. This included CaMKII T286L in Cos-7 cell extracts [29], T286A and T286P partially purified from CHO cells (with the T286P mutant exhibiting mildly increased non-stimulated basal activity) [30], and T286A produced in a reticulocyte *in vitro* translation system (additionally describing that a T286D mutant mimics the phosphorylated state by resulting in almost the same level of autonomous activity) [31]. Again, in all cases, this appears to be based on individual kinase preparations. However, in hippocampal extracts from three wild type and three T286A mice, no significant difference in Ca^2+^/CaM-stimulated CaMKII activity was detected either [13]. When an approximate 25% contribution of CaMKIIβ in the extracts is subtracted, the mean activity in the wild type extracts was ∼1.1fold higher compared to T286A extracts. However, based on the observed variability, only effects larger than ∼1.6fold could have reached significance, while the mild ∼1.2–1.3fold effect reported here could not have been resolved.

CaMKII made “autonomous” by T286 phosphorylation is significantly (∼3–6fold) further stimulated by Ca^2+^/CaM [18], indicating an only partial opening of the CaMKII inhibitory gate by T286 phosphorylation, with significant further opening by additional Ca^2+^/CaM binding. The results of this study indicate that the more complete opening of the CaMKII inhibitory gate by Ca^2+^/CaM binding alone may, *vice versa*, also be further increased by additional T286 phosphorylation, albeit much more mildly (with only ∼1.2–1.3fold increased kinase activity). Several partial crystal structures of CaMKII in its auto-inhibited basal state are now available [21], [55]–[57], and are consistent with the inhibitory gate (physically provided by interactions of the regulatory domain with the kinase domain) mainly blocking substrate access and to a lesser extent also reducing ATP binding (for review see [5]). A partial crystal structure of an activated state of CaMKII provides information about structural shifts in the kinase domain and about the Ca^2+^/CaM interaction [57], consistent with mutually exclusive CaM-binding and T305/306 phosphorylation. However, while it is clear that the positioning of the regulatory domain must change in the different activation states of CaMKII, the actual position relative to the kinase domains within a holoenzyme remains to be elucidated for both the stimulated and autonomous states of CaMKII.

## References

[1] Erondu NE, Kennedy MB (1985). Regional distribution of type II Ca2+/calmodulin-dependent protein kinase in rat brain.. J Neurosci.

[2] Tobimatsu T, Fujisawa H (1989). Tissue-specific expression of four types of rat calmodulin-dependent protein kinase II mRNAs.. J Biol Chem.

[3] Bayer KU, Lohler J, Schulman H, Harbers K (1999). Developmental expression of the CaM kinase II isoforms: ubiquitous gamma- and delta-CaM kinase II are the early isoforms and most abundant in the developing nervous system.. Brain Res Mol Brain Res.

[4] Lisman J, Schulman H, Cline H (2002). The molecular basis of CaMKII function in synaptic and behavioural memory.. Nat Rev Neurosci.

[5] Hudmon A, Schulman H (2002). Neuronal CA2+/calmodulin-dependent protein kinase II: the role of structure and autoregulation in cellular function.. Annu Rev Biochem.

[6] Lee YS, Silva AJ (2009). The molecular and cellular biology of enhanced cognition.. Nat Rev Neurosci.

[7] Malinow R, Schulman H, Tsien RW (1989). Inhibition of postsynaptic PKC or CaMKII blocks induction but not expression of LTP.. Science.

[8] Silva AJ, Stevens CF, Tonegawa S, Wang Y (1992). Deficient hippocampal long-term potentiation in alpha-calcium-calmodulin kinase II mutant mice.. Science.

[9] Derkach V, Barria A, Soderling TR (1999). Ca^2+^/calmodulin-kinase II enhances channel conductance of alpha-amino-3-hydroxy-5-methyl-4-isoxazolepropionate type glutamate receptors.. Proc Natl Acad Sci U S A.

[10] Hayashi Y, Shi SH, Esteban JA, Piccini A, Poncer JC (2000). Driving AMPA receptors into synapses by LTP and CaMKII: requirement for GluR1 and PDZ domain interaction.. Science.

[11] Opazo P, Labrecque S, Tigaret CM, Frouin A, Wiseman PW (2010). CaMKII Triggers the Diffusional Trapping of Surface AMPARs through Phosphorylation of Stargazin.. Neuron.

[12] Kristensen AS, Jenkins MA, Banke TG, Schousboe A, Makino Y (2011). Mechanism of Ca2+/calmodulin-dependent kinase II regulation of AMPA receptor gating.. Nat Neurosci.

[13] Giese KP, Fedorov NB, Filipkowski RK, Silva AJ (1998). Autophosphorylation at Thr286 of the alpha calcium-calmodulin kinase II in LTP and learning.. Science.

[14] Buard I, Coultrap SJ, Freund RK, Lee YS, Dell'Acqua ML (2010). CaMKII “autonomy” is required for initiating but not for maintaining neuronal long-term information storage.. J Neurosci.

[15] Miller SG, Kennedy MB (1986). Regulation of brain type II Ca2+/calmodulin-dependent protein kinase by autophosphorylation: a Ca2+-triggered molecular switch.. Cell.

[16] Lou LL, Lloyd SJ, Schulman H (1986). Activation of the multifunctional Ca2+/calmodulin-dependent protein kinase by autophosphorylation: ATP modulates production of an autonomous enzyme.. Proc Natl Acad Sci U S A.

[17] Schworer CM, Colbran RJ, Soderling TR (1986). Reversible generation of a Ca2+-independent form of Ca2+(calmodulin)-dependent protein kinase II by an autophosphorylation mechanism.. J Biol Chem.

[18] Coultrap SJ, Buard I, Kulbe JR, Dell'Acqua ML, Bayer KU (2010). CaMKII autonomy is substrate-dependent and further stimulated by Ca2+/calmodulin.. J Biol Chem.

[19] De Koninck P, Schulman H (1998). Sensitivity of CaM kinase II to the frequency of Ca2+ oscillations.. Science.

[20] Bayer KU, De Koninck P, Schulman H (2002). Alternative splicing modulates the frequency-dependent response of CaMKII to Ca(2+) oscillations.. EMBO J.

[21] Chao LH, Stratton MM, Lee IH, Rosenberg OS, Levitz J (2011). A mechanism for tunable autoinhibition in the structure of a human Ca2+/calmodulin- dependent kinase II holoenzyme.. Cell.

[22] Hanson PI, Meyer T, Stryer L, Schulman H (1994). Dual role of calmodulin in autophosphorylation of multifunctional CaM kinase may underlie decoding of calcium signals.. Neuron.

[23] Rich RC, Schulman H (1998). Substrate-directed function of calmodulin in autophosphorylation of Ca2+/calmodulin-dependent protein kinase II.. J Biol Chem.

[24] Bradshaw JM, Hudmon A, Schulman H (2002). Chemical quenched flow kinetic studies indicate an intraholoenzyme autophosphorylation mechanism for Ca2+/calmodulin-dependent protein kinase II.. J Biol Chem.

[25] Kwiatkowski AP, Shell DJ, King MM (1988). The role of autophosphorylation in activation of the type II calmodulin-dependent protein kinase.. J Biol Chem.

[26] Katoh T, Fujisawa H (1991). Autoactivation of calmodulin-dependent protein kinase II by autophosphorylation.. J Biol Chem.

[27] Ishida A, Kitani T, Fujisawa H (1996). Evidence that autophosphorylation at Thr-286/Thr-287 is required for full activation of calmodulin-dependent protein kinase II.. Biochim Biophys Acta.

[28] Tzortzopoulos A, Torok K (2004). Mechanism of the T286A-mutant alphaCaMKII interactions with Ca2+/calmodulin and ATP.. Biochemistry.

[29] Hanson PI, Kapiloff MS, Lou LL, Rosenfeld MG, Schulman H (1989). Expression of a multifunctional Ca2+/calmodulin-dependent protein kinase and mutational analysis of its autoregulation.. Neuron.

[30] Ohsako S, Nakazawa H, Sekihara S, Ikai A, Yamauchi T (1991). Role of threonine-286 as autophosphorylation site for appearance of Ca2(+)-independent activity of calmodulin-dependent protein kinase II alpha subunit.. J Biochem.

[31] Fong YL, Taylor WL, Means AR, Soderling TR (1989). Studies of the regulatory mechanism of Ca2+/calmodulin-dependent protein kinase II. Mutation of threonine 286 to alanine and aspartate.. J Biol Chem.

[32] Bayer KU, De Koninck P, Leonard AS, Hell JW, Schulman H (2001). Interaction with the NMDA receptor locks CaMKII in an active conformation.. Nature.

[33] Singla SI, Hudmon A, Goldberg JM, Smith JL, Schulman H (2001). Molecular characterization of calmodulin trapping by calcium/calmodulin-dependent protein kinase II.. J Biol Chem.

[34] Coultrap SJ, Bayer KU, Mukai H (2012). Ca^2+^/Calmodulin-Dependent Protein Kinase II (CaMKII).. Neuromethods: Protein Kinase Technologies: Humana Press.

[35] Vest RS, Davies KD, O'Leary H, Port JD, Bayer KU (2007). Dual Mechanism of a Natural CaMKII Inhibitor.. Mol Biol Cell.

[36] Colbran RJ (1993). Inactivation of Ca2+/calmodulin-dependent protein kinase II by basal autophosphorylation.. J Biol Chem.

[37] Hanson PI, Schulman H (1992). Inhibitory autophosphorylation of multifunctional Ca2+/calmodulin-dependent protein kinase analyzed by site-directed mutagenesis.. J Biol Chem.

[38] Vest RS, O'Leary H, Coultrap SJ, Kindy MS, Bayer KU (2010). Effective post-insult neuroprotection by a novel Ca(2+)/calmodulin-dependent protein kinase II (CaMKII) inhibitor.. J Biol Chem.

[39] Hashimoto Y, Schworer CM, Colbran RJ, Soderling TR (1987). Autophosphorylation of Ca2+/calmodulin-dependent protein kinase II. Effects on total and Ca2+-independent activities and kinetic parameters.. J Biol Chem.

[40] Patton BL, Miller SG, Kennedy MB (1990). Activation of type II calcium/calmodulin-dependent protein kinase by Ca2+/calmodulin is inhibited by autophosphorylation of threonine within the calmodulin-binding domain.. J Biol Chem.

[41] Elgersma Y, Fedorov NB, Ikonen S, Choi ES, Elgersma M (2002). Inhibitory autophosphorylation of CaMKII controls PSD association, plasticity, and learning.. Neuron.

[42] Meyer T, Hanson PI, Stryer L, Schulman H (1992). Calmodulin trapping by calcium-calmodulin-dependent protein kinase.. Science.

[43] Glazewski S, Giese KP, Silva A, Fox K (2000). The role of alpha-CaMKII autophosphorylation in neocortical experience-dependent plasticity.. Nat Neurosci.

[44] Hardingham N, Glazewski S, Pakhotin P, Mizuno K, Chapman PF (2003). Neocortical long-term potentiation and experience-dependent synaptic plasticity require alpha-calcium/calmodulin-dependent protein kinase II autophosphorylation.. J Neurosci.

[45] Wilbrecht L, Holtmaat A, Wright N, Fox K, Svoboda K (2010). Structural plasticity underlies experience-dependent functional plasticity of cortical circuits.. J Neurosci.

[46] Gustin RM, Shonesy BC, Robinson SL, Rentz TJ, Baucum AJ, 2nd (2011). Loss of Thr286 phosphorylation disrupts synaptic CaMKIIalpha targeting, NMDAR activity and behavior in pre-adolescent mice.. Mol Cell Neurosci.

[47] Radwanska K, Medvedev NI, Pereira GS, Engmann O, Thiede N (2011). Mechanism for long-term memory formation when synaptic strengthening is impaired.. Proc Natl Acad Sci U S A.

[48] Yamagata Y, Kobayashi S, Umeda T, Inoue A, Sakagami H (2009). Kinase-dead knock-in mouse reveals an essential role of kinase activity of Ca2+/calmodulin-dependent protein kinase IIalpha in dendritic spine enlargement, long-term potentiation, and learning.. J Neurosci.

[49] Otmakhov N, Griffith LC, Lisman JE (1997). Postsynaptic inhibitors of calcium/calmodulin-dependent protein kinase type II block induction but not maintenance of pairing-induced long-term potentiation.. J Neurosci.

[50] Chen HX, Otmakhov N, Strack S, Colbran RJ, Lisman JE (2001). Is persistent activity of calcium/calmodulin-dependent kinase required for the maintenance of LTP?. J Neurophysiol.

[51] Sanhueza M, Fernandez-Villalobos G, Stein IS, Kasumova G, Zhang P (2011). Role of the CaMKII/NMDA receptor complex in the maintenance of synaptic strength.. J Neurosci.

[52] Ikeda A, Okuno S, Fujisawa H (1991). Studies on the generation of Ca2+/calmodulin-independent activity of calmodulin-dependent protein kinase II by autophosphorylation. Autothiophosphorylation of the enzyme.. J Biol Chem.

[53] Colbran RJ (2004). Targeting of calcium/calmodulin-dependent protein kinase II.. Biochem J.

[54] Merrill MA, Chen Y, Strack S, Hell JW (2005). Activity-driven postsynaptic translocation of CaMKII.. Trends Pharmacol Sci.

[55] Rosenberg OS, Deindl S, Sung RJ, Nairn AC, Kuriyan J (2005). Structure of the autoinhibited kinase domain of CaMKII and SAXS analysis of the holoenzyme.. Cell.

[56] Chao LH, Pellicena P, Deindl S, Barclay LA, Schulman H (2010). Intersubunit capture of regulatory segments is a component of cooperative CaMKII activation.. Nat Struct Mol Biol.

[57] Rellos P, Pike AC, Niesen FH, Salah E, Lee WH (2010). Structure of the CaMKIIdelta/calmodulin complex reveals the molecular mechanism of CaMKII kinase activation.. PLoS Biol.

